# Preparative Purification of Bioactive Compounds from* Flos Chrysanthemi Indici* and Evaluation of Its Antiosteoporosis Effect

**DOI:** 10.1155/2016/2587201

**Published:** 2016-11-03

**Authors:** Jia Li, Xiaosheng Lin, Yuping Zhang, Weicai Liu, Xiaohui Mi, Jiquan Zhang, Jiansheng Su

**Affiliations:** ^1^Department of Prosthodontics, School & Hospital of Stomatology, Tongji University, Shanghai Engineering Research Center of Tooth Restoration and Regeneration, Shanghai, China; ^2^Engineering Research Center of Modern Preparation Technology of TCM, Ministry of Education, Shanghai University of Traditional Chinese Medicine, Shanghai, China

## Abstract

To understand the material basis and underlying molecular machinery of antiosteoporosis activity of the* Flos Chrysanthemi Indici* (FCI), the consequences of ethanol extract on the bone loss in mice induced due to ovariectomy (OVX) was evaluated. Also, the antiosteoporosis fraction obtained from the FCI ethanol extract was isolated and purified using a preparative high-speed countercurrent chromatography (HSCCC). The* in vitro* impact of the compounds was investigated on osteoblast proliferation and differentiation. The results revealed that ethyl acetate fraction with robust* in vivo* antiosteoporosis activity was obtained. The important compounds purified by HSCCC using gradient elution system included acacetin, apigenin, luteolin, and linarin. The four compounds enhanced the differentiation and proliferation of osteoblasts in MC3T3-E1 cells. They also augmented the mRNA levels of runt-related transcription factor 2 (Runx2), osteocalcin (OCN), osteopontin (OPN), and type I collagen (COL I). The AKT signaling pathway was also activated in MC3T3-E1 cells by the four compounds. The present study demonstrated that the antiosteoporosis effects of FCI did not depend on a single component, and HSCCC efficiently isolated and purified the antiosteoporosis bioactive compounds from FCI.

## 1. Introduction

Osteoporosis is characterized as a metabolic bone disease, wherein the bone microarchitecture is deteriorated due to disruption of bone formation and resorption [1, 2]. Currently, several therapeutics are available for the treatment of osteoporosis. Clinically, the use of inhibitors for bone resorption (e.g., bisphosphonates and calcitonin) has been proved to be efficient and fruitful against osteoporosis [3]; however, their long-term usage may exhibit adverse reactions. For example, the extended use of bisphosphonate elevates the risk of osteonecrosis of the jaw [4]. Moreover, the inhibitors of bone resorption moderately induce the bone formation. Hormone replacement therapy prevents osteoporosis or positively affects the bone formation during the treatment of the disease [5, 6]. Hence, secondary metabolites from natural plants for increasing bone density remain an alternative approach for managing osteoporosis.

*Flos Chrysanthemi Indici* (FCI), an important species of Asteraceae family, possesses anti-inflammatory, antioxidative, and antihypertension properties [7–9] and has thus been traditionally used in Chinese herbal medicines. A recent report suggested that the Chrysanthemums ethanol extract could promote osteogenic activities of osteoblastic cells [10]. Our previous study demonstrated that FCI extract treatment can effectively prevent ovariectomy- (OVX-) induced bone loss in mice [11]. However, the underlying molecular basis of the antiosteoporosis activity of FCI has not been fully understood. Therefore, identification and purification of the bioactive fractions from FCI, for the treatment of osteoporosis, are vital for further investigation of its pharmacological mechanism.

HSCCC is a liquid-liquid partition chromatographic technique, which has been widely utilized for the segregation of naturally occurring compounds [12–14]. The lack of a solid stationary phase renders HSCCC advantageous over the traditional liquid-solid separation methods. The disadvantages of these methods include irreversible adsorption of the sample onto the solid matrix, deactivation and/or contamination of the stationary phase, poor sample recovery, and difficulty in scaling up the production of the samples [15]. Previously, we also described the usage of HSCCC in the isolation and purification of four flavonoids from Herba Salviae Plbeiae [16]. Therefore, HSCCC could serve as an excellent method for rapid separation and purification of potentially applicative bioactive products from FCI.

The present study describes the effect of the bioactive fractions from the ethanol extract of FCI on the bone loss induced by OVX in mice. Also, the preparative separation of the four compounds from the bioactive fractions has been established using HSCCC in a stepwise elution mode with optimized operation parameters. Moreover, the effect of the four compounds on osteoblastic proliferation and differentiation was investigated* in vitro*. Hitherto, a systematic evaluation of the ethanol extract of FCI against osteoporosis and HSCCC separation of antiosteoporosis active compounds has not been described.

## 2. Materials and Methods

### 2.1. Chemicals and Reagents

HPLC grade acetonitrile and analytical grade organic solvents, as well as ammonium acetate for sample preparation and HSCCC separation, were obtained from (Sinopharm Chemical Reagent Co. Ltd., Shanghai, China). Ultrapure water was achieved by RU-B water system (Shanghai Tauto Biotech Co. Ltd., Shanghai, China) and filtered through 0.45 *μ*m before use.

*Flos Chrysanthemi Indici* was bought from the medicinal material market (Haozhou, Anhui, China). Cell Counting Kit-8 (CCK-8) was obtained from Dojindo Molecular Technologies (Minato-ku, Tokyo, Japan). Alizarin red-S and calcium colorimetric assay kit were procured from Sigma-Aldrich (St. Louis, MO, USA). The primary antibodies targeting phospho-AKT (Ser473; #4060) and AKT (#4691) were procured from Cell Signaling Technology (Danvers, MA, USA) and Runx2 antibody from Abcam (ab76956; Cambridge, UK).

### 2.2. Apparatus

The preparative HSCCC instrument, TBE-300C (Shanghai Tauto Biotech), was serially connected with three multilayer coil separation columns comprising 1.9 mm internal tubing diameter of the tubing and 305 mL total volume as well as a 20 mL loop for sample loading. The *β*-value of the multilayer coil was in the range of 0.59–0.75 at the internal and external terminals, respectively. The revolution speed of the apparatus was regulated at 0–900 rpm. The HSCCC system, Dionex HPLC system, and the NMR spectrophotometer were identical to those described previously [16].

### 2.3. Crude Sample Preparation

**Figure 1:**
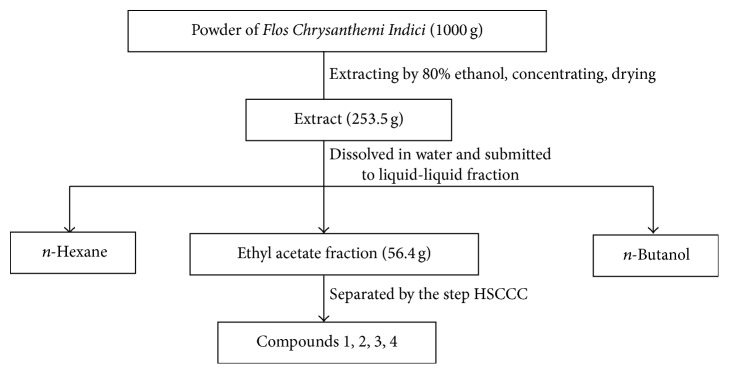
The separation scheme of* Flos Chrysanthemi Indici*.

FCI (1000 g) was homogeneously powdered by a mill and ultrasonically solubilized in in 5 L of 80% ethanol at 60°C for 2 h (repeated thrice). The extracts were pooled, filtered, and dried under reduced pressure at 55°C, yielding 253.5 g, which was then solubilized in water. Subsequently, the extraction was carried out with *n*-hexane followed by ethyl acetate and *n*-butanol in the same order, which yielded 9.7 g *n*-hexane extract (FCI-H), 56.4 g ethyl acetate extract (FCI-E), and 31.7 g *n*-butanol extract (FCI-B) (Figure 1).

### 2.4. Bioassay of Variable Fractions for OVX-Induced Osteoporosis

The fractions obtained from the ethanol extract were subjected to antiosteoporosis assay on OVX-induced osteoporosis in mice. Forty female C57/BL6 mice aged 8 weeks (21.2 ± 1.3 g) were obtained from the Shanghai Laboratory Animal Center (SLAC, Shanghai, China), and the experiments were approved by the Institutional Animal Care and Use Committee of Shanghai Tongji University. Animals in the OVX group were ovariectomized bilaterally, whereas the control group mice were sham-operated. One week after operation, the OVX mice were orally administered with 100 mg/kg *n*-hexane (FCI-H), ethyl acetate (FCI-E), or *n*-butanol (FCI-B) fraction daily. Subsequent to 8-week treatment, a micro-computed tomography (micro-CT) system (*μ*CT50, Scanco Medical, Bassersdorf, Switzerland) at a resolution of 10 *μ*m was employed for scanning the microstructural indices of the distal femurs including the bone mineral density (BMD) of the trabecular bone, bone volume/tissue volume (BV/TV), trabecular space (Tb.Sp), and trabecular number (Tb.N).

### 2.5. HSCCC Protocol

According to the results of the experiments described in Section 2.4, FCI-E was selected for HSCCC separation which consisted of the two-phase solvent system: *n*-hexane-chloroform-methanol-water (0.5 : 4 : 3 : 2, v/v/v/v) and chloroform-methanol-water (4 : 3 : 2, v/v/v) for gradient elution. Saturated ammonium acetate aqueous solution with a volume equivalent to 0.5% of the total was added as a demulsifier. A separating funnel was utilized to ensure complete equilibration of the solvent mixture. The two phases separated at room temperature were sonicated for 20 min to degas them. 500 mg crude sample solubilized in 20 mL of the mobile phase was loaded on the preparative HSCCC for separation.

**Figure 2:**
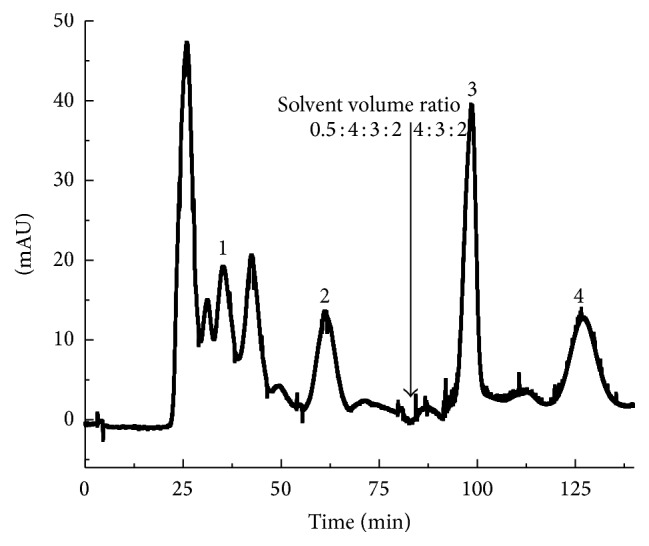
HSCCC chromatograms of ethyl acetate fraction from* Flos Chrysanthemi Indici*. Separation by gradient solvent system *n*-hexane-chloroform-methanol-water (0.5 : 4 : 3 : 2, v/v/v/v) to chloroform-methanol-water (4 : 3 : 2, v/v/v) using TBE-300C with the following conditions: elution mode: head-to-tail; flow rate: 5.0 mL/min; revolution speed: 850 rpm; column temperature: 25°C; *S*_*f*_ was 69.6%; stationary phase: upper phase; mobile phase: lower phase; detection: 340 nm. Peaks: acacetin (1), apigenin (2), luteolin (3), and linarin (4).

HSCCC was performed at 25°C column temperature and 850 rpm rotary speed. The flow rate and solvent gradients were altered according to the *K*_*D*_ values of the target compounds.  Figure 2 demonstrated that the separation begins with *n*-hexane-chloroform-methanol-water (0.5 : 4 : 3 : 2, v/v/v/v,) solvent system. Compounds 1 and 2 were eluted at 80 min (dotted line in Figure 2), following which the mobile phase was switched to chloroform-methanol-water (4 : 3 : 2, v/v/v), that is, the lower phase of the solvent system, for the elution of compounds 3 and 4. The HSCCC peak fractions were monitored at 340 nm, manually collected based on the chromatogram, and dried under reduced pressure for subsequent purity analysis by HPLC. The volume collected from the column after the completion of the separation was used for enumerating the stationary phase retained on the column in correlation with the total column capacity.

### 2.6. HPLC Analysis of FCI-E and Purity Determination of the Peak Fraction

The FCI-E and HSCCC peak fractions were analyzed by HPLC at 25°C. Acetonitrile-0.1% H_3_PO_4_ aq was used as a gradient mobile phase for acetonitrile for 0–60 min, 15–80% mode at a flow rate of 1.0 mL/min. The effluent was monitored at 335 nm, and the structure of each peak fraction was resolved by ^1^H and ^13^C NMR spectra.

### 2.7. Cell Culture

MC3T3-E1 cells (ATCC; Manassas, VA, USA) were cultured in phenol red-free *α*-MEM (Invitrogen, Carlsbad, CA, USA) supplemented with 10% charcoal-stripped FBS (Hyclone, Logan, UT, USA), 1% penicillin, and 1% streptomycin at 37°C in a 5% CO_2_ humidified incubator. Osteogenic differentiation was induced in the cells by culturing them in complete *α*-MEM with 10 mM *β*-glycerophosphate and 50 *μ*g/mL ascorbic acid.

### 2.8. Assay of Four Compounds for Osteoblast Proliferation, Alkaline Phosphatase (ALP) Activity, and Mineralized Matrix

MC3T3-E1 cells were seeded and cultured for 24 h in 96-well plates at a density of 4 × 10^3^ cells/well. Subsequently, the cells were treated with the test agent at concentrations of 0.1, 1, and 10 *μ*M for 48 h. 10 *μ*L of the CCK-8 reagent was then added to each well and incubated for 1 h. The rate of cell proliferation was assessed by measuring the absorbance at 450 nm on a microplate reader.

The cells were incubated with the test agent at concentration of 1 *μ*M in 24-well plates. After 7 or 10 days of osteogenic induction, the supernatants from the cell lysates were collected. The alkaline phosphatase activity was detected using p-nitrophenyl phosphate (pNPP) (Beyotime, Jiangsu, China) as the substrate by incubating the samples and the substrate at 37°C for 20 min. After the reaction had been quenched, the absorbance was measured at 405 nm. The total protein concentration was estimated by a protein assay reagent (Bio-Rad, Hercules, CA, USA) according to the manufacturer's instructions. The ALP activity was normalized to the total protein.

The cells were seeded and cultured in 24-well plates at a density of 1.5 × 10^5^ cells/well. Subsequently, the cells were treated with the test agent at concentration of 1 *μ*M. After 21 days of osteogenic induction, the calcium deposition staining and quantitative analysis were detected using Alizarin red-S and a calcium ion assay kit, respectively. Briefly, after washing three times with cold PBS and fixing with 75% ethyl alcohol, the cells were stained with Alizarin red-S (40 mM, pH 4.2), and the pictures of calcium deposition were photographed under a Nikon microscope. Moreover, the quantitative analysis of calcium concentration was measured by colorimetric detection at 570 nm. The calcium concentration was normalized to the total protein.

### 2.9. Assay of Four Compounds for Osteogenesis-Related Gene Expression

MC3T3-E1 cells were cultured in 24-well plates for 24 h, followed by treatment with the test agent at concentrations of 1 *μ*M for 7 or 10 days. Total RNA was isolated using RNeasy Mini Kits (Qiagen, Valencia, CA, USA), and cDNA was synthesized by reverse transcription kit (TaKaRa Biotechnology, Otsu, Japan). The mRNA levels of type I collagen (COL-I), osteopontin (OPN), osteocalcin (OCN), and runt-related transcription factor 2 (Runx2) were evaluated on ABI 7500 Sequencing Detection System (Applied Biosystems, Foster City, CA, USA) using SYBR Premix Ex-Taq kit (TaKaRa Biotechnology). The RT-qPCR reaction was as follows: 50°C, 2 min; initial denaturation at 95°C, 10 min, followed by 40 cycles of 95°C for 15 s, 60°C for 30 s, and 72°C for 30 s. The real-time data was assimilated in the final extension step. All reactions were in triplicate and data were represented as the fold changes relative to the control.*β-Actin* served as an endogenous control. The primer sequences for specific targets were listed in Table 1.

### 2.10. Assay of Four Compounds for Osteogenesis-Related Protein Expression

MC3T3-E1 cells were incubated with different components from FCI in 6-well plates for Western blotting analysis. The soluble fraction of the cells was collected using RIPA lysis buffer (Thermo, DE), and the protein concentration was estimated as described above. 20 *μ*g of the total protein was resolved by SDS-PAGE (10% gel) and blotted onto PVDF membrane (Pall, Port Washington, NY, USA). The membrane was subjected to blocking and then incubated with primary antibodies, rabbit anti-phospho-AKT (Ser473), rabbit anti- AKT, mouse anti-Runx2 at 1 : 1000, and mouse polyclonal anti-*β*-actin (sc-47778; Santa Cruz Biotechnology, Inc., Santa Cruz, CA, USA) at 1 : 2000. Subsequently, the blots were probed with goat anti-rabbit or anti-mouse HRP-conjugated secondary antibody (sc-2004 or sc-2005) at 1 : 5000. The immunobands were visualized by the ECL detection system (Santa Cruz Biotechnology).

### 2.11. Statistical Analysis

All the experiments were independently repeated at least thrice, and data were represented as mean ± SD. The results between different groups were compared using one-way analysis of variance (ANOVA). *P* < 0.05 denotes a statistically significant difference. SPSS 17.5 was used for analysis.

## 3. Results and Discussion

### 3.1. Bioassay of Different Fractions for OVX-Induced Osteoporosis

**Figure 3:**
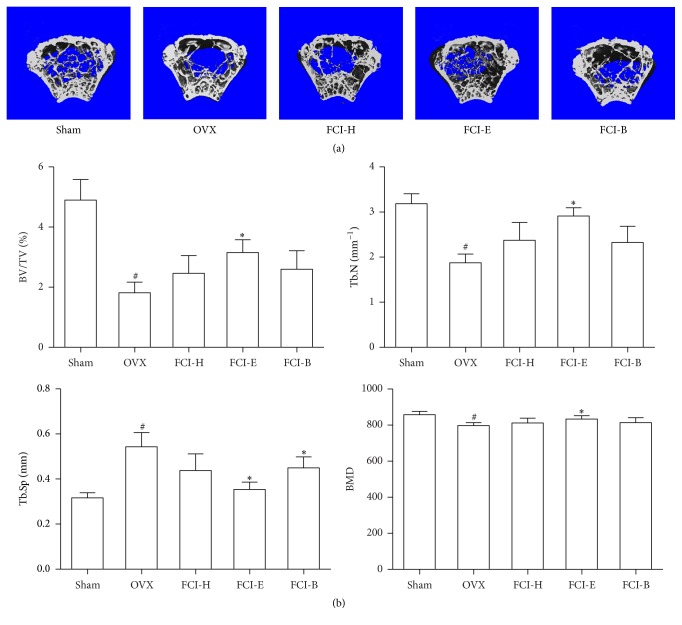
Bioassay of different fractions from FCI for ovariectomized (OVX) mice. OVX mice were orally given 100 mg/kg *n*-hexane (FCI-H), ethyl acetate (FCI-E), or *n*-butanol (FCI-B) fraction for 8 weeks. (a) *μ*CT scan of the metaphyseal distal femurs in each group. (b) The microstructural indices were quantified from data obtained by the *μ*CT, including bone volume/tissue volume (BV/TV), trabecular number (Tb.N), trabecular space (Tb.Sp), and bone mineral density (BMD). The results are expressed as the means ± SD, *n* = 8/group. ^*∗*^ *P* < 0.05 as compared with OVX; ^#^ *P* < 0.05 as compared with sham-operated group.

The *μ*CT examination of OVX-induced osteoporosis in ovariectomized mice showed a massive loss of bone at metaphyseal distal femurs (Figure 3). The quantitative analysis of the bone mineral content revealed significantly reduced BMD, BV/TV, and Tb.N and substantially increased Tb.Sp. The* in vivo* effect of fractions of FCI-E (100 mg/kg) displayed that the trabecular bone microarchitecture was well preserved in OVX mice. Moreover, FCI-E caused an increase in BMD compared to the control OVX group. The treatment of OVX mice with FCI-H or FCI-B (100 mg/kg) did not affect the BMD and the bone mineral content significantly. Therefore, the FCI-E fraction from the ethanol extract of FCI was used in the downstream experiments.

### 3.2. HSCCC Separation of Bioactive Compounds

The two-phase solvent systems comprising various ratio volumes of *n*-hexane-chloroform-methanol-water were tested for an efficient resolution of the bioactive components.  Table 2 showed the *K*_*D*_ values of the target compounds in the different solvent systems. The *K*_*D*_ values were low for compounds 1 and 2 in systems 1 and 2 and hence could not be well separated. However, in the presence of system 3, compounds 1, 2, 3, and 4 could be separated, with *K*_*D*_ values 0.55, 0.86, 1.27, and 3.38, respectively. However, compound 1 was not completely distinct from the preceding peak, which was overcome with system 4, wherein compound 1 peak was distinctly resolved. Consequently, compound 4 was retained on the column for an extended time due to a large *K*_*D*_ value (9.05). The results suggested that all the compounds could not be rapidly and conveniently separated using a single solvent system.

Therefore, a stepwise HSCCC elution mode simultaneously separated the compounds with large *K*_*D*_ values [17]. This method was previously utilized for successfully separating four flavonoids from* Herba Salviae Plbeiae* [16]. Herein, *n*-hexane-chloroform-methanol-water (0.5 : 4 : 3 : 2, v/v/v/v) system was first used for the elution of compounds 1 and 2, followed by the second solvent system comprising the mobile phase, chloroform-methanol-water (4 : 3 : 2, v/v/v), for the elution of the remaining components.

Similarly, a number of HSCCC separation experiments were performed for optimizing the operational parameters, including the column temperature, resolution speed, and the mobile phase flow rate for preparative separations. Initially, the optimal column temperature, revolution speed, and the solvent's flow rate were 25°C, 850 rpm, and 5.0 mL/min, respectively. The sample size encountered an enormous limitation with the destruction of hydrodynamic equilibrium caused by the emulsification between the two phases. About 100 mL of the stationary phase was eluted upon 300 mg sample injection, leading to the decline in the retention rate of the stationary phase from 69.6% to 37.0%. Thus, the sample loading capacity was limited, and it reduced the peaks' resolution on HSCCC. The addition of electrolytes was the appropriate strategy to enhance the coagulation of droplets. Ultimately, 0.5% saturated ammonium acetate, a volatile salt, of the total volume was added to eliminate emulsification to avoid the time-consuming desalting process and increase the sample loading capacity from 300 mg to 500 mg.

### 3.3. Structure Identification of the HSCCC Peaks

**Figure 4:**
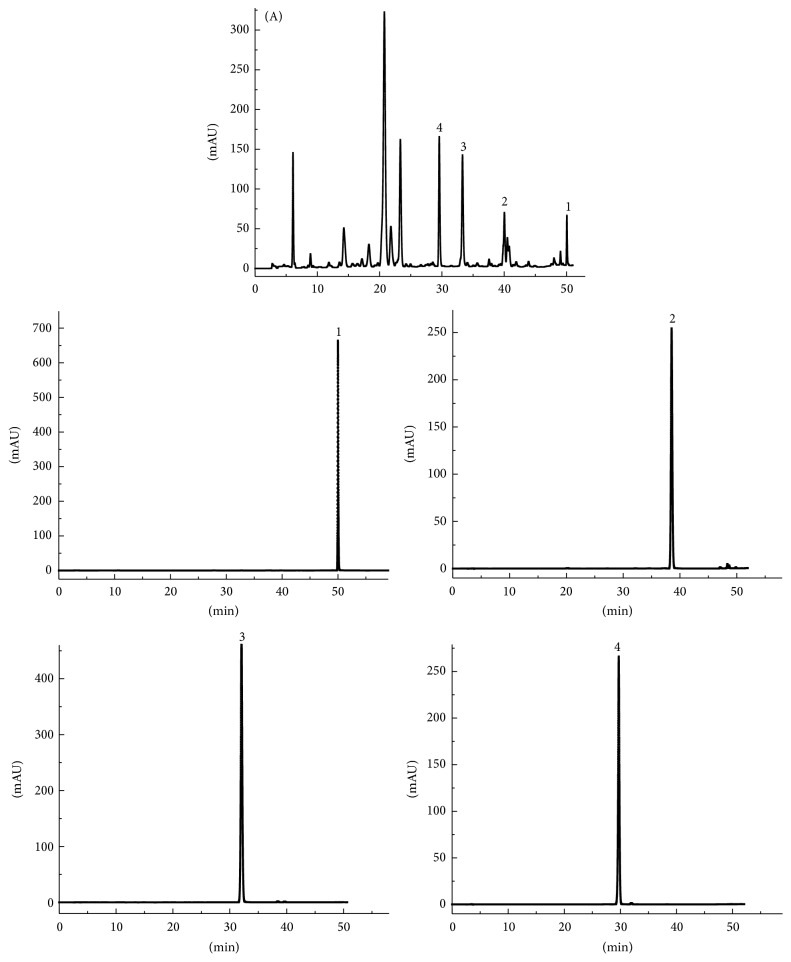
HPLC chromatograms of ethyl acetate fraction from* Flos Chrysanthemi Indici* and HSCCC peak fractions. (A) Ethyl acetate fraction from* Flos Chrysanthemi Indici*; (1–4) purified HSCCC peak fractions 1–4 in Figure 3. Conditions: column, Boston crest C18, (250 mm × 4.6 mm, ID 5 *μ*m); column temperature, 25°C; mobile phase, acetonitrile, and 0.1% H_3_PO_4_ aq at the gradient (acetonitrile 0–14 min, 16–21%; 14–32 min, 21–34%; 32–42 min, 34–42%; 42–60 min, and 42–80 min); flow rate, 1.0 mL/min; detection, 326 nm.

One-step HSCCC preparative separation of 500 mg crude sample resulted in 6.7 mg compound 1, 13.7 mg compound 2, 33.4 mg compound 3, and 32.8 mg compound 4 with the purities of 95.1%, 98.0%, 98.5%, and 98.6%, respectively, as revealed by HPLC analysis (Figure 4).

^1^H and ^13^C NMR spectra determined the chemical structures of the peak fractions.

*Compound 1*. Yellow powder, ^1^H NMR (500 MHz, DMSO-d6) *δ*: 12.91 (1H, s, OH-5), 10.82 (1H, s, OH-7), 8.03 (2H, d, *J* = 9.0 Hz, H-2′, 6′), 7.11 (2H, d, *J* = 9.0 Hz, H-3′, 5′), 6.85 (1H, s, H-3), 6.50 (1H, d, *J* = 2.1 Hz, H-8), 6.20 (1H, d, *J* = 2.1 Hz, H-6), 3.86 (3H, s, OCH_3_); ^13^C NMR (150 MHz, DMSO-d6) *δ*: 181.7 (C-4), 164.1 (C-7), 163.2 (C-2), 162.2 (C-4′), 161.4 (C-9), 157.3 (C-5), 128.2 (C-2′, 6′), 122.7 (C-1′), 114.5 (C-3′, 5′), 103.7 (C-10), 103.5 (C-3), 98.8 (C-6), 94.0 (C-8), 55.5 (OCH_3_).

*Compound 2*. Yellow powder, ^1^H NMR (500 MHz, DMSO-d6) *δ*: 14.72 (1H, s, H-5), 7.98 (2H, d, *J* = 8.6 Hz, H-2′, 6′), 7.24 (2H, d, *J* = 8.6 Hz, H-3′, 5′), 6.89 (1H, s, H-3), 6.85 (1H, d, *J* = 1.6 Hz, H-8), 6.71 (1H, d, *J* = 1.6 Hz, H-6); ^13^C NMR (150 MHz, DMSO-d6) *δ*: 165.5 (C-2), 103.3 (C-3), 184.8 (C-4), 163.2 (C-5), 94.5 (C-6), 164.9 (C-7), 100.8 (C-8), 158.5 (C-9), 105.2 (C-10), 123.3 (C-1′), 128.4 (C-2′), 116.7 (C-3′), 164.0 (C-4′), 117.2 (C-5′), 127.3 (C-6′).

*Compound 3*. Light yellow powder, ^1^H NMR (500 MHz, DMSO-d6) *δ*: 7.94 (1H, s, H-2′), 7.44 (1H, d, *J* = 8.3 Hz, H-6′), 7.33 (1H, d, *J* = 8.3 Hz, H-5′), 6.83 (1H, s, H-3), 6.75 (2H, s, H-6, 8); ^13^C NMR (150 MHz, DMSO-d6) *δ*: 164.3 (C-2), 103.9 (C-3), 182.9 (C-4), 160.1 (C-5), 100.8 (C-6), 166.7 (C-7), 95.7 (C-8), 164.1 (C-9), 105.9 (C-10), 119.6 (C-1′), 115.5 (C-2′), 149.3 (C-3′), 153.7 (C-4′), 118.2 (C-5′), 122.3 (C-6′).

*Compound 4*. White powder, ^1^H NMR (500 MHz, DMSO-d6) *δ*: 7.96 (2H, d, *J* = 8.5 Hz, H-2′, 6′), 6.94 (2H, d, *J* = 8.5 Hz, H-3′, 5′), 6.88 (1H, d, *J* = 2.5 Hz, H-8), 6.83 (1H, s, H-3), 6.44 (1H, d, *J* = 2.5 Hz, H-6), 5.32 (1H, d, *J* = 6.0 Hz, H-1′′), 5.26 (1H, d, *J* = 2.0 Hz, H-1′′′); ^13^C NMR (150 MHz, DMSO-d6) *δ*: 164.1 (C-2), 163.1 (C-7), 160.5 (C-9), 103.3 (C-3), 182.1 (C-4), 157.1 (C-5), 95.2 (C-8), 99.7 (C-6), 104.5 (C-10), 121.4 (C-1′), 127.8 (C-2′, 6′), 114.8 (C-3′, 5′), 162.5 (C-4′), 55.4 (OCH_3_), 101.5 (C-1′′), 73.2 (C-2′′), 76.1 (C-3′′), 70.9 (C-4′′), 76.6 (C-5′′), 66.7 (C-6′′), 100.9 (C-1′′′), 70.2 (C-2′′′), 69.7 (C-3′′′), 72.2 (C-4′′′), 68.4 (C-5′′′), 17.9 (C-6′′′).

**Figure 5:**
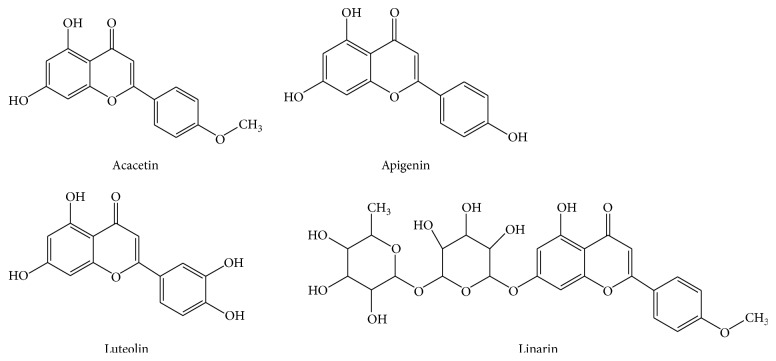
Chemical structures of the four flavonoids from* Flos Chrysanthemi Indici*.

Compared with the data in the literature [18, 19], compounds 1, 2, 3, and 4 were identified as acacetin, apigenin, luteolin, and linarin (Figure 5).

### 3.4. Effect of the Four Compounds on Osteoblast Proliferation, ALP Activity, and Mineralized Matrix

**Figure 6:**
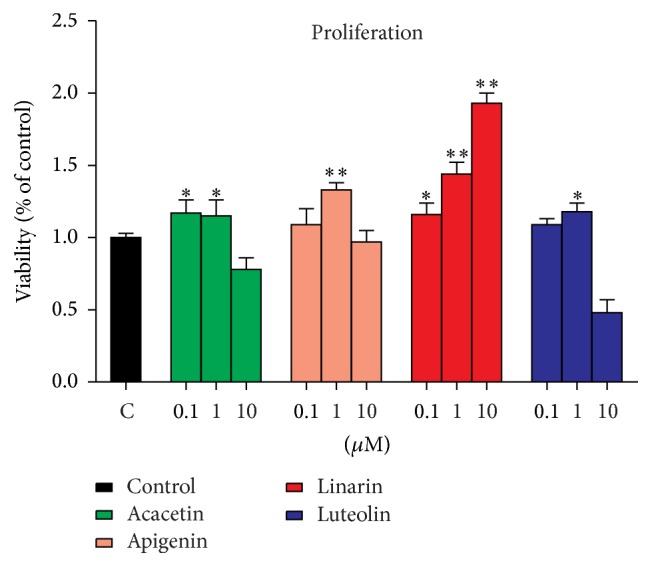
Effect of the four compounds on cell proliferation of MC3T3-E1 cells. The cell viability was measured by CCK-8 assay. All the compounds significantly affected the cell proliferation after 48 h incubation. Data are represented as the means ± SD of three independent experiments. ^*∗*^ *P* < 0.05 and ^*∗∗*^ *P* < 0.01 as compared with the control.

Skeleton is composed of several cell types that are continuously evolved for the integration of the structure in a global mineral and nutrient homeostasis [20]. Osteoblasts are vital for bone formation. Hence, the effect of the four compounds on osteoblast proliferation was assessed in MC3T3-E1 cells by CCK-8 assay. All the compounds significantly affected the cell proliferation after 48 h incubation (Figure 6). Acacetin, apigenin, and luteolin showed significant cell proliferation activity as compared to the control at the concentrations of 0.1 and 1 *μ*M but showed cell toxicity at 10 *μ*M in MC3T3-E1 cells. Linarin showed the most significant cell proliferation activity in a dose-dependent manner after 48 h treatment.

**Figure 7:**
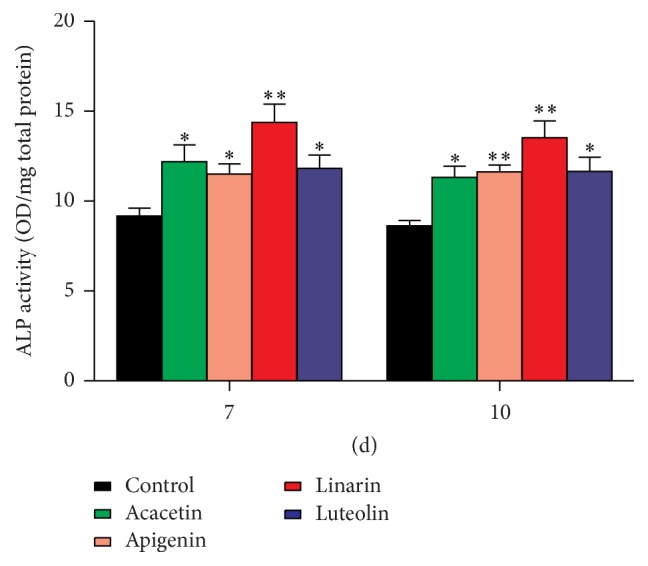
Effect of the four bioactive compounds on alkaline phosphatase (ALP) activity of MC3T3-E1 cells. The ALP activity was found to be significantly increased by the four bioactive compounds at a concentration of 1 *μ*M after 7 or 10 days of incubation. ALP activity was assessed using a commercial ALP kit. Data are represented as the means ± SD of three independent experiments. ^*∗*^ *P* < 0.05 as compared with the control.

ALP is a key osteoblastic phenotype marker contributing towards a high phosphate concentration for mineral deposition [21, 22]. The ALP activity was found to be significantly increased by the four bioactive compounds at a concentration of 1 *μ*M after 7 or 10 days of incubation. Moreover, among the four compounds, linarin showed the most significant ALP activity (Figure 7).

**Figure 8:**
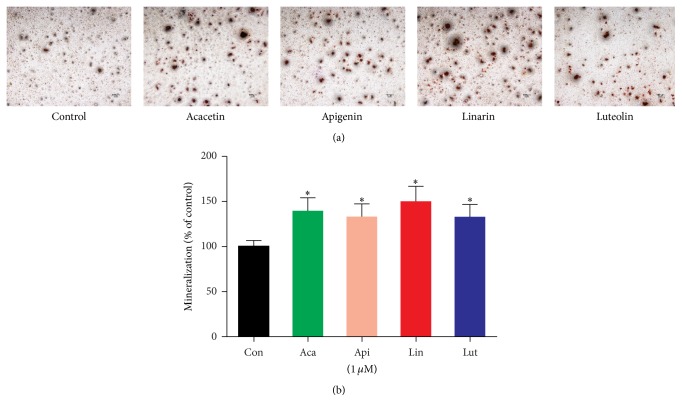
Effect of the four compounds on mineralization of MC3T3-E1 cells. (a) After treatment with test agent at concentration of 1 *μ*M for 21 days, the calcium deposition was assessed by Alizarin red-S staining (×4). (b) Calcium concentration analysis demonstrated that the four compounds increased the mineralization of MC3T3-E1 cells. Data are represented as the means ± SD of three independent experiments. ^*∗*^ *P* < 0.05 as compared with the control.

The degree of mineralized matrix in cells is an important marker for the analysis of the osteogenic potential of the four compounds. The formation of calcified deposition was found to be significantly increased by the four bioactive compounds at a concentration of 1 *μ*M after 21 days of incubation. The Alizarin red-S staining in four compounds-treated groups was more intense than that in the control group (Figure 8(a)). Moreover, the quantitative analysis of mineralized matrix demonstrated that the calcium concentration in four compounds-treated groups was higher than that in the control group (Figure 8(b)). Results of proliferation, ALP activity, and mineralized matrix suggest that the four compounds at the appropriate concentration promote the osteoblast proliferation and differentiation.

### 3.5. Effect of the Four Compounds on Osteogenesis-Related Gene and Protein Expression

**Figure 9:**
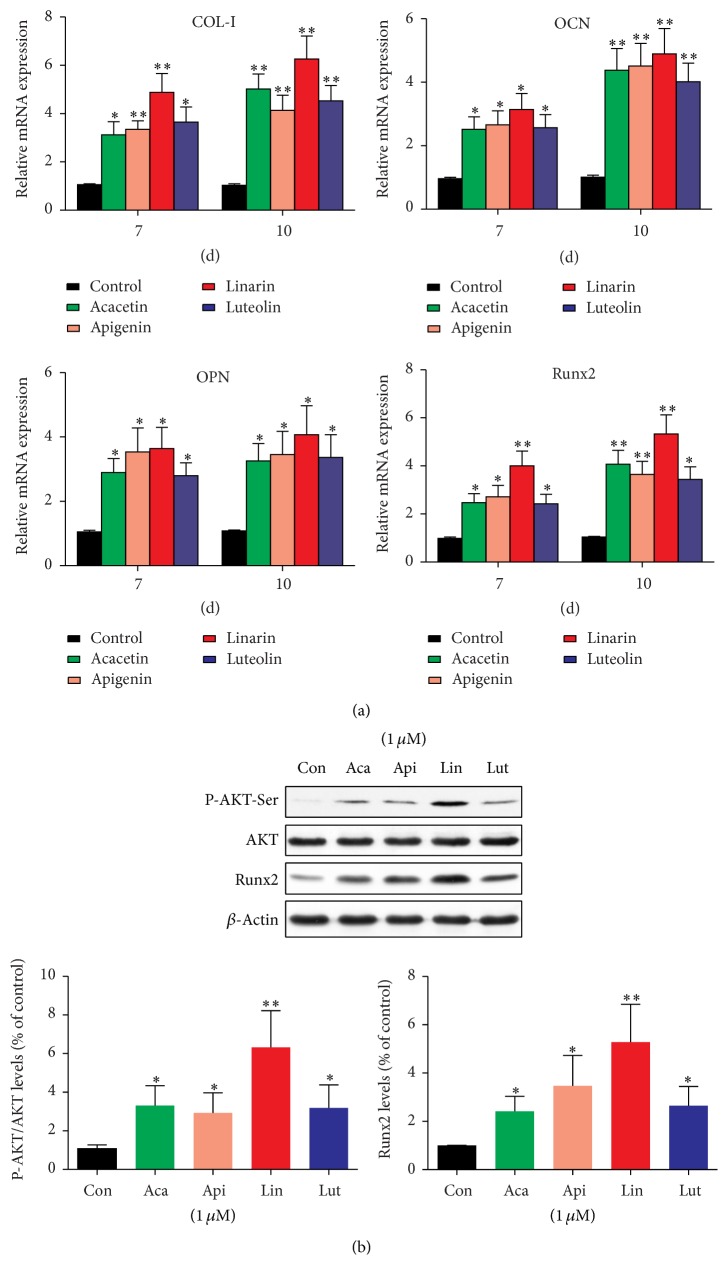
Effect of four compounds on osteogenesis-related gene and protein expression. (a) MC3T3-E1 cells were pretreated with 1 *μ*M test agent and were then induced to undergo osteogenesis for 7 or 10 days. Levels of t type I collagen (COL-I), osteopontin (OPN), osteocalcin (OCN), and runt-related transcription factor 2 (Runx2) mRNA expression were analyzed by RT-qPCR. (b) Western blot analysis of AKT phosphorylation and Runx2 expression after treatment of MC3T3-E1 cells with the indicated concentration of test agent. Data are represented as the means ± SD of three independent experiments. ^*∗*^ *P* < 0.05 and ^*∗∗*^ *P* < 0.01 as compared with the control.

Osteoblast differentiation is regulated by a large number of osteogenic differentiation molecular markers such as COL-I, OPN, and OCN. COL-I is a vital organic component in the extracellular matrix (ECM) of the bone that regulates the early osteoblast differentiation and consociates the cell surface integrins with other ECM proteins [23]. OPN, an intermediate marker of osteogenic differentiation, is related to the maturation stage of osteoblasts during matrix assembly [24]. OCN, secreted by osteoblasts, regulates the bone formation at a later stage [25]. Our data demonstrated that osteoblastic cells treatment with apigenin and luteolin at a concentration of 1 *μ*M significantly increased the transcriptional level of COL-I, OPN, and OCN (Figure 9(a)). Similar concentrations of these two compounds have been used, and our findings are in agreement with the other experiments [26–28]. In a previous study, we reported that linarin extracted from FCI enhanced the osteoblast differentiation in MC3T3-E1 cells in a dose-dependent manner [11]. Furthermore, as best known, herein, we demonstrate for the first time that acacetin, apigenin, and luteolin extracted from FCI upregulated the gene expression of COL-I, OPN, and OCN, thereby promoting osteoblast differentiation.

AKT signaling pathway is pivotal for the regulation of osteogenic differentiation and bone remolding [29, 30]. To address the role of the AKT signaling pathway in the induction of osteogenic differentiation by FCL extract, we investigated the phosphorylation of AKT under the treatment of acacetin, apigenin, luteolin, and linarin, respectively. It was shown that the four different components at a concentration of 1 *μ*M significantly phosphorylated AKT at Ser473 in MC3T3-E1 cells (Figure 9(b)). Runx2, a critical transcriptional factor for osteoblast differentiation, is regulated by AKT signaling pathway as demonstrated by defective intramembranous ossification in AKT knockout animals [31, 32]. Therefore, Runx2 expression in MC3T3-E1 cells was elucidated in the presence of the four compounds. Runx2 was found to be activated at both mRNA and protein levels, indicating that acacetin, apigenin, luteolin, and linarin extracted from FCI induce osteogenic differentiation via the AKT-Runx2 pathway. Among the four different compounds, linarin showed remarkable osteogenic differentiation activities. Although future studies are necessitated to fully define the precise molecular mechanism and elucidate the effect of the four compounds on bone metabolism* in vivo*, our findings suggest that FCI extract is a potential natural therapeutic for osteoporosis.

## 4. Conclusions

In the present study, a competent fractionation method was developed for the first time for the identification and purification of bioactive compounds from the ethanol extract of* Flos Chrysanthemi Indici* and termed as HSCCC. The ethyl acetate fraction harbored the intact trabecular bone microarchitecture of OVX mice. One-step HSCCC separation of ethyl acetate fraction resulted in acacetin (6.7 mg), apigenin (13.7 mg), luteolin (33.4 mg), and linarin (32.8 mg), with >95% purity, according to HPLC. Furthermore, the four compounds promoted osteoblastic proliferation and differentiation, out of which, linarin exhibited remarkable osteogenic differentiation activities. The results from the present study suggested that ethyl acetate fraction was a natural alternative for the treatment of osteoporosis and that its activity did not depend on a single component. Also, the bioactive compounds from FCI were efficiently purified by HSCCC.

## Figures and Tables

**Table 1:** Specific primers for RT-PCR analysis.

Gene	Forward	Reverse
COL-I	5′-GAGCGGAGTACTGGATCG-3′	5′-GCTTCTTTTCCTTGGGGTT-3′
OPN	5′-GATCAGGACAACAACGGAAAGG-3′	5′-GCTGGCTTTGGAACTTGCTT-3′
OCN	5′-GAGGACCATCTTTCTGCTCACTCT-3′	5′-TTATTGCCCTCCTGCTTGGA-3′
Runx2	5′-GCACAAACATGGCCAGATTCA-3′	5′-AAGCCATGGTGCCCGTTAG-3′
*β*-Actin	5′-TCTGCTGGAAGGTGGACAGT-3′	5′-CCTCTATGCCAACACAGTGC-3′

**Table 2:** The *K* values of the target compounds at different two-phase solvent systems.

Solvent system	*K* values			
	Compound 1	Compound 2	Compound 3	Compound 4
(1) Chloroform-methanol-water (4 : 2 : 2)	0.21	0.29	1.08	2.15
(2) Chloroform-methanol-water (4 : 3 : 3)	0.25	0.31	1.11	2.06
(3) Chloroform-methanol-water (4 : 3 : 2)	0.55	0.86	1.27	3.38
(4) *n*-Hexane-chloroform-methanol-water (0.5 : 4 : 3 : 2)	0.62	1.13	2.29	9.05

Compound 1: acacetin; Compound 2: apigenin; Compound 3: luteolin; Compound 4: linarin.

## Abbreviations

FCI: * Flos Chrysanthemi Indici*
OVX: Ovariectomy
HSCCC: High-speed countercurrent chromatography
CCK-8: Cell counting kit-8
*K*_*D*_: Distribution constants
NMR: Nuclear magnetic resonance
ALP: Alkaline phosphatase
OPN: Osteopontin
OCN: Osteocalcin
COL-I: Type I collagen
Runx2: Runt-related transcription factor 2
BMD: Bone mineral density
BV/TV: Bone volume/tissue volume
Tb.Sp: Trabecular space
Tb.N: Trabecular number.
